# Efficacy and safety of needle-free jet injection of self-crosslinking hyaluronic acid for facial fine wrinkles and skin texture: 12-week clinical outcomes in Asian women

**DOI:** 10.3389/fmed.2026.1832590

**Published:** 2026-07-14

**Authors:** Kyu-Ho Yi, Yerin Park, Soo-Yeon Lee, Han Earl Lee, Atchima Suwanchinda

**Affiliations:** 1 You and I Clinic, Seoul, Republic of Korea; 2 Medical Research Inc., Wonju, Republic of Korea; 3 Opening Plastic Surgery Clinic, Seoul, Republic of Korea; 4 Department of Dermatology, Chulabhorn Royal Academy, Bangkok, Thailand

**Keywords:** facial rejuvenation, fine wrinkles, needle-free jet injection, self-crosslinking hyaluronic acid, skin texture

## Abstract

**Introduction:**

Facial aging involves progressive deterioration of the superficial dermal matrix, leading to fine wrinkles, surface irregularity, and reduced skin elasticity. Conventional hyaluronic acid (HA) formulations vary in their ability to achieve uniform superficial dermal dispersion and sustained tissue effects, and needle injections may lead to inconsistent depth and localized pooling. This study evaluated changes in facial skin texture and fine wrinkles following delivery of self-crosslinking HA formulation containing N-hyaluronoyl 5-hydroxydopamine through a needle-free jet injection.

**Methods:**

Thirty women aged 40–55 years with Fitzpatrick skin types III–IV and visible fine wrinkles underwent three treatment sessions at 4-week intervals. Outcomes included wrinkle and texture scores derived from three-dimensional imaging in the malar and pericommissural regions, investigator-rated fine wrinkle severity, patient-reported improvement, and safety assessment.

**Results:**

At week 12, malar wrinkle and texture scores decreased by 8.7 and 7.2 points, respectively, and pericommissural wrinkle and texture scores decreased by 7.5 and 4.9 points, respectively, with statistically significant improvements across all quantitative imaging parameters (all *p* < 0.01). Investigator-rated fine wrinkle severity significantly improved from 7.28 ± 1.18 to 4.32 ± 1.36 (*p* < 0.01). Overall, 60% of participants reported moderate to marked improvement, and treatment-related reactions were mild and transient.

**Discussion:**

The concordant improvements in three-dimensional imaging and investigator-rated outcomes suggest that needle-free jet delivery of self-crosslinking HA may improve fine wrinkles and skin texture through superficial dermal modulation. Further controlled studies with longer follow-up are needed to confirm durability and comparative efficacy.

## Introduction

Fine wrinkles are among the earliest and most visible manifestations of facial skin aging. Unlike deep static folds associated with volumetric loss or structural descent, fine wrinkles primarily arise from cumulative alterations within the superficial dermal matrix, including reduced water-binding capacity, collagen fibril fragmentation, elastin degradation, and diminished mechanical resilience of the papillary and upper reticular dermis. These changes result in surface irregularity and loss of smooth skin texture, particularly in dynamic facial regions such as the perioral and malar areas (1–3).

Hyaluronic acid (HA) plays a central role in maintaining dermal hydration and viscoelasticity. Injectable HA-based products for facial rejuvenation are generally categorized into non-crosslinked HA formulations, chemically crosslinked HA fillers, and hybrid or composite materials designed to improve skin quality rather than volume (1, 4, 5). Non-crosslinked HA exhibits favorable diffusion within the superficial dermis and can transiently increase dermal thickness and skin quality; however, its rapid enzymatic degradation limits the durability of clinical effects, especially for sustained fine wrinkle improvement (6, 7).

Chemically crosslinked HA fillers were developed to enhance molecular stability and tissue persistence, and intradermal injection of BDDE-crosslinked HA has been shown to induce wrinkle reduction and dermal remodeling (8, 9). Nevertheless, increased elastic modulus, cohesivity, and particle integrity associated with chemical crosslinking may restrict uniform dispersion within superficial dermal layers, where fine wrinkles originate (5, 10, 11). In addition, experimental toxicity studies have raised considerations regarding the cellular effects of chemical crosslinking agents in delicate dermal compartments (12, 13).

To address the trade-off between diffusibility and durability, self-crosslinking HA systems have been introduced. These formulations are designed to achieve in situ molecular stabilization while preserving flexibility for homogeneous superficial dermal dispersion, thereby prolonging tissue residence without forming discrete filler boluses. As such, self-crosslinking HA is conceptually suited for fine wrinkle modulation and global skin texture improvement rather than volumetric correction (14).

Delivery method is another critical determinant of outcome in superficial dermal rejuvenation. Conventional needle-based intradermal injections are susceptible to variability in depth and localized pooling. In contrast, needle-free jet injection systems generate high-velocity microjets capable of consistent intradermal penetration and lateral dispersion. Sonographic and comparative studies have demonstrated that jet-based delivery can improve depth consistency and distribution while reducing procedure-related discomfort and needle-associated adverse events (15–17).

Therefore, the present study was designed to evaluate the efficacy and safety of a self-crosslinking hyaluronic acid formulation delivered via a needle-free jet injection system for improving fine wrinkles and skin texture across the full face, using objective 3D imaging alongside clinical assessments.

## Materials and methods

### Study design and participants

This single-arm, non-randomized interventional study was designed to assess the effects of needle-free jet injection of a self-crosslinking hyaluronic acid formulation on facial fine wrinkles and skin texture. Participants who underwent all three treatment sessions at 4-week intervals and completed the final follow-up assessment at week 12 were included in the analysis. Participants were treated at the dermatology department of You and I Clinic, Seoul, Republic of Korea.

Female participants aged 40–55 years with Fitzpatrick skin types III–IV were included if they had clinically evident facial fine wrinkles, defined as an investigator-rated fine wrinkle severity score of 5 or higher on a 10-point scale. Only female participants were included to minimize sex-related heterogeneity in facial skin characteristics and aging patterns. Exclusion criteria included pregnancy or lactation, active inflammatory or infectious facial skin conditions, prior energy-based facial procedures or injectable treatments within 6 months before enrollment, and known hypersensitivity to hyaluronic acid–based products.

Written informed consent was obtained from all participants. This study protocol was approved by the Public Institutional Bioethics Committee designated by the Ministry of Health and Welfare, Republic of Korea (Approval No. P01-202511-01-062) and conducted in accordance with the Declaration of Helsinki. The study was reported in accordance with the Transparent Reporting of Evaluations with Nonrandomized Designs (TREND) guidelines.

### Materials

The injectable material used in this study was a self-crosslinking hyaluronic acid formulation (HyLua N.42 for Reclock; Baz Biomedic, Seoul, Republic of Korea). This formulation undergoes self-crosslinking *in vivo* in response to local oxygen exposure and physiological blood pH following administration, allowing molecular stabilization without the use of conventional chemical crosslinking agents.

HyLua N.42 contains N-hyaluronoyl 5-hydroxydopamine as the hyaluronic acid–based component, with a hyaluronic acid molecular weight of approximately 1.0 MDa. The absence of chemical crosslinkers such as 1,4-butanediol diglycidyl ether (BDDE) or divinyl sulfone (DVS) distinguishes this formulation from conventional crosslinked hyaluronic acid products. The formulation also includes trehalose to support hydration by reducing moisture loss, along with disodium phosphate and potassium phosphate as buffering components to maintain formulation stability.

For intradermal delivery, HyLua N.42 was supplied as a sterile injectable solution and administered using a needle-free jet injection system. CureJet (Baz Biomedic, Seoul, Republic of Korea) is a needle-free intradermal delivery system that employs electromechanical jet injection technology to generate high-velocity liquid microjets for transdermal delivery without the use of a solid needle. Upon electrical activation, an electromagnetic field drives a piston forward, transferring kinetic energy through a membrane to the liquid formulation and resulting in rapid ejection through a narrow nozzle. As the liquid exits the nozzle, it accelerates to form a focused microjet capable of penetrating the epidermis and reaching the dermal layer, where it disperses without creating a sharp puncture tract (15–17).

**FIGURE 1:**
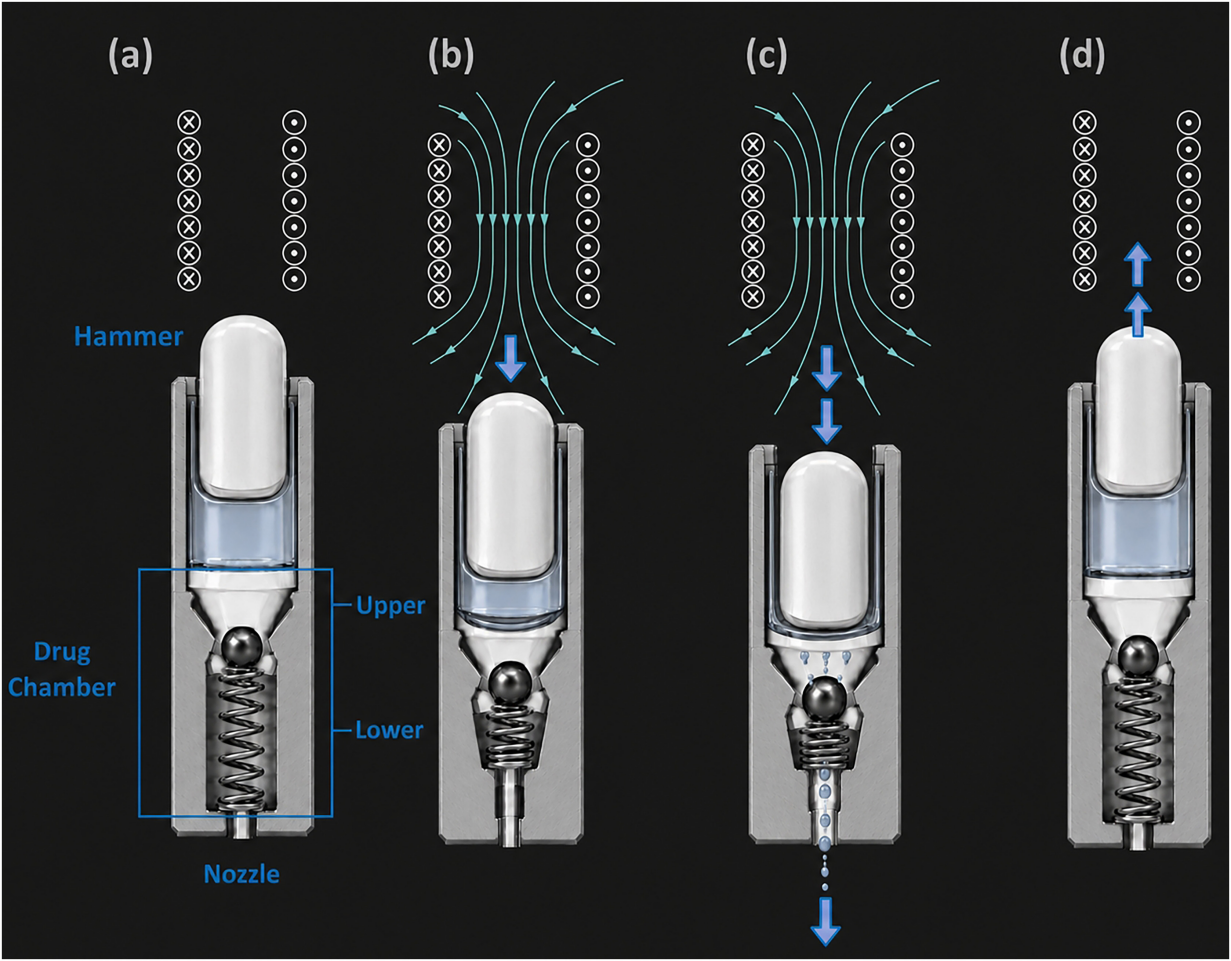
Schematic representation of the operating sequence of the needle-free jet injection system. **(a)** System state before activation. **(b)** Activation of the electromagnetic coil generates a magnetic field that drives linear displacement of the piston, transmitting mechanical force to the interchamber membrane. **(c)** Opening of the nozzle enables ejection of the liquid formulation as a focused microjet. **(d)** Upon completion of delivery, the nozzle closes and the chamber is refilled in preparation for the next injection cycle.

The system supports repeated microjet delivery with adjustable power settings ranging from level 1 to 20, with each actuation dispensing a controlled microvolume. By eliminating needle penetration, CureJet minimizes mechanical tissue injury and puncture-related trauma associated with conventional injection methods. The operating mechanism of the device is illustrated schematically in Figure 1.

### Treatment protocol

All participants received treatment involving the upper, mid, and lower facial regions using a self-crosslinking hyaluronic acid formulation delivered via a needle-free jet injection system. Treatments were administered in three sessions at 4-week intervals (weeks 0, 4, and 8). In each treatment session, a total volume of 2.5 mL was delivered per participant, corresponding to approximately 4,500 jet injections distributed across the treated facial regions. The injection volume was precisely controlled by the device, dispensing 0.56 μL of HA per injection point. To ensure uniform coverage, adjacent injection points were spaced at 2–3 mm intervals. Needle-free delivery was conducted using both distance and contact modes. Distance mode was first applied over the upper, mid, and lower facial regions to provide broad full-face superficial dermal coverage. In this mode, the nozzle was positioned perpendicular to the skin surface at approximately 90° with a nozzle-to-skin clearance of >15 mm, and two passes were performed across the treated facial areas. After full-face distance-mode treatment, contact mode was additionally applied as a focused delivery step only to areas with prominent fine wrinkles, including the malar cheek and perioral/pericommissural regions. In the contact mode, the nozzle was positioned at approximately 45–60° relative to the skin surface, maintaining a nozzle-to-skin clearance of >2 mm, and a single focused pass was performed over these focal areas. These delivery modes were performed under the selected machine settings, with the expected injection depth targeted to the superficial and upper reticular dermis, corresponding to a depth of 0.6–1.2 mm from the epidermis, to optimally address fine wrinkles without deep tissue pooling.

All participants received a topical anesthetic cream containing 2.5% lidocaine and 2.5% prilocaine (EMLA; Aspen Pharmacare, Durban, South Africa) for 30 min prior to each treatment session. Participants were instructed to maintain their usual skincare routine and to refrain from initiating any new treatments intended to alter pigmentation or modify skin condition during the study period.

### Quantitative assessment

Objective wrinkle analysis was performed using a three-dimensional imaging system (Antera 3D; Miravex, Dublin, Ireland). Quantitative wrinkle parameters were evaluated in predefined regions of interest, including the malar (cheek) region and pericommissural skin adjacent to the oral commissures.

Measured parameters included wrinkle score and surface roughness. All images were acquired under standardized lighting and positioning conditions, and the same regions of interest were analyzed at baseline and at the final follow-up visit.

### Qualitative assessment

Dermatologist-rated severity scores were obtained by two independent dermatologist evaluators blinded to the treatment process, using a validated 10-point photonumeric grading scale adapted for Asian facial wrinkles (18). Fine wrinkles were defined as visible superficial linear creases of the facial skin, distinct from deep folds primarily associated with volume loss or tissue descent. Fine wrinkles, skin texture, and pigmentation uniformity were each graded from 0 to 10, with 0 indicating absence of the feature and 10 indicating severe, pathological presentation.

Standardized clinical photographs were obtained at baseline and week 12. All photographs were anonymized and randomized prior to evaluation, and investigators were unaware of the treatment time point and whether images were obtained before or after treatment.

Patient-reported outcomes included overall improvement assessed at the final follow-up visit using a 5-point ordinal scale based on perceived percentage improvement (1 = none; 2 = 1–24%; 3 = 25–49%; 4 = 50–74%; 5 = 75–100%).

### Safety assessments

Safety assessments involved monitoring and documentation of treatment-related adverse events throughout the study period. Adverse events of interest included localized erythema, edema, pain, bruising, and any unexpected skin reactions at the treated areas.

Procedural pain was assessed immediately after each treatment session using a visual analog scale (VAS) ranging from 0 to 10, where 0 represented no pain and 10 represented the worst imaginable pain.

### Statistical analysis

Continuous variables are presented as mean ± standard deviation, and categorical variables are presented as number and percentage. Quantitative imaging parameters and investigator-rated severity scores were assessed at baseline and week 12; therefore, within-participant changes between the two time points were analyzed using paired *t*-tests. Normality was assessed using the Shapiro–Wilk test, and Wilcoxon signed-rank tests were considered when the normality assumption was not satisfied.

Procedural pain scores were measured repeatedly after each treatment session and were analyzed using one-way repeated-measures analysis of variance (RM-ANOVA). When the assumption of sphericity was violated, Greenhouse–Geisser correction was applied. Categorical outcomes, including patient-reported improvement and adverse events, were summarized descriptively. A two-sided *p* < 0.05 was considered statistically significant. Statistical analyses were performed using IBM SPSS Statistics for Windows, version 29.0 (IBM Corp., Armonk, NY, United States).

The analyzed sample size was determined based on the total number of eligible participants who completed the full 12-week protocol. To verify the adequacy of the sample size, a sensitivity analysis demonstrated that 30 analyzable participants provided 80% statistical power to detect a within-participant standardized mean difference of 0.53 in paired comparisons at a two-sided alpha level of 0.05. Therefore, the analyzed cohort was considered sufficient for detecting within-participant changes in fine wrinkles and skin texture.

## Results

A total of 34 participants were initially assessed, all of whom met the eligibility criteria and were enrolled. Four participants were lost to follow-up before the Week 12 assessment due to personal scheduling conflicts unrelated to the treatment. No participants discontinued the study because of adverse events. Therefore, 30 participants completed all treatment sessions and the final follow-up assessment at week 12 and were included in the final analysis. The mean age of the analyzed population was 47.3 ± 4.2 years. All participants were female with Fitzpatrick skin types III–IV.

### Quantitative outcomes

Three-dimensional imaging demonstrated statistically significant reductions in wrinkle and texture scores from baseline to final follow-up within predefined regions. In the malar region, the wrinkle score changed by −8.7 and the texture score by −7.2, while in the pericommissural region, the wrinkle score changed by −7.5 and the texture score by −4.9 (all *p* < 0.01). Corresponding values are provided in Table 1.

**TABLE 1:** Wrinkle and texture scores assessed by 3D imaging system.

Region	Parameter	Baseline	Week 12	Change (Δ)	*p*-value
Malar region	Wrinkle score	33.6 ± 2.3	24.9 ± 2.6	−8.7	< 0.01
	Texture score	27.8 ± 1.7	20.6 ± 1.9	−7.2	< 0.01
Pericommissural region	Wrinkle score	38.9 ± 2.8	31.4 ± 3.1	−7.5	< 0.01
	Texture score	31.7 ± 2.1	26.8 ± 2.3	−4.9	< 0.01

### Qualitative outcomes

**FIGURE 2:**
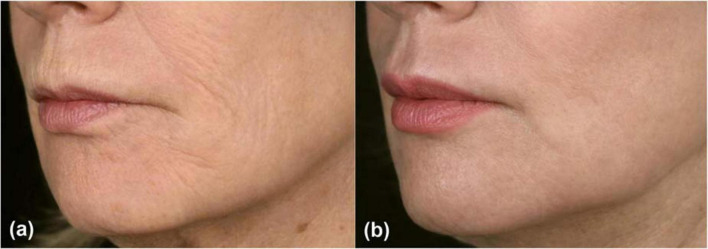
Before-and-after photographs of a 48-year-old woman treated with three sessions of needle-free jet injection of a self-crosslinking HA formulation. **(a)** Baseline image of the perioral and lower facial region showing fine linear wrinkles and uneven skin surface. **(b)** Week 12 image of the same region showing changes in the appearance of superficial fine lines and skin surface texture.

**FIGURE 3:**
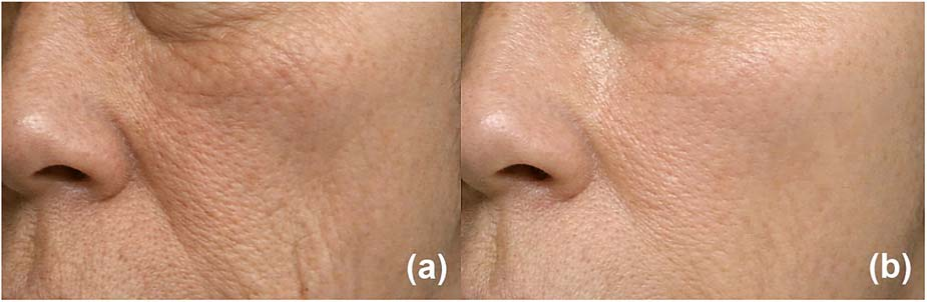
Before-and-after photographs of a 53-year-old woman treated with a self-crosslinking HA delivered via needle-free jet injection. **(a)** Baseline image of the malar region demonstrating fine wrinkles and surface irregularity. **(b)** Week 12 image of the same region showing changes in the appearance of superficial wrinkles and skin surface texture.

Changes in investigator-rated severity scores for fine wrinkles, skin texture, and pigmentation uniformity from baseline to week 12 are presented in Table 2. Statistically significant improvements were observed across all assessed clinical parameters following treatment. Figures 2, 3 illustrate visible improvement in fine wrinkles and skin texture following treatment with self-crosslinking hyaluronic acid delivered via needle-free jet injection.

**TABLE 2:** Investigator-rated severity scores at baseline and week 12.

Parameter	Baseline (Mean ± SD)	Week 12 (Mean ± SD)	*p*-value
Fine wrinkles	7.28 ± 1.18	4.32 ± 1.36	< 0.01
Skin texture	6.14 ± 1.44	4.41 ± 1.22	< 0.01
Pigmentation uniformity	6.72 ± 1.61	5.38 ± 1.47	< 0.01

Patient-reported improvement was assessed using a 5-point ordinal scale. Based on this scale, 18 participants (60.0%) reported moderate to marked improvement. An additional 9 participants (30.0%) reported mild to moderate improvement, while 3 participants (10.0%) reported minimal or no improvement (Table 3).

**TABLE 3:** Patient-reported overall improvement at final follow-up (5-point ordinal scale, *n* = 30).

Score	Definition (perceived improvement)	Number of participants (%)
5	75–100% improvement	7 (23.3)
4	50–74% improvement	11 (36.7)
3	25–49% improvement	9 (30.0)
2	1–24% improvement	2 (6.7)
1	No improvement	1 (3.3)

### Safety outcomes

Procedural pain assessed using the visual analog scale showed low to moderate discomfort. Mean VAS scores were 3.2 ± 1.2, 3.0 ± 1.1, and 2.9 ± 1.0 for the first, second, and third treatment sessions, respectively, with no significant increase across sessions.

Reported adverse events were limited to transient, localized reactions at the treatment sites. The most commonly reported events included mild erythema (*n* = 14, 46.7%), transient edema (*n* = 9, 30.0%), and mild procedural pain (*n* = 11, 36.7%), all of which resolved spontaneously within several hours to days. No serious adverse events, delayed inflammatory reactions, or treatment discontinuations were observed during the study period.

## Discussion

### Main findings

In this study, three sessions of needle-free jet injection of self-crosslinking hyaluronic acid improved objective and clinical measures of facial fine wrinkles and skin texture. On three-dimensional imaging, wrinkle scores decreased by 8.7 points in the malar region and 7.5 points in the pericommissural region, while texture scores decreased by 7.2 and 4.9 points, respectively. Investigator-rated fine wrinkle severity improved from 7.28 ± 1.18 at baseline to 4.32 ± 1.36 at Week 12. In addition, 90.0% of participants reported minimal to marked improvement, and treatment-related reactions were mild and transient. These findings indicate that the treatment produced measurable improvements in fine wrinkles and skin texture over the 12-week follow-up period, with acceptable tolerability.

### Facial aging

Facial skin aging is driven by gradual changes in cutaneous architecture and tissue composition, with concurrent alterations in deeper soft tissue and skeletal support that together affect surface appearance. Prior studies have demonstrated that visible facial aging reflects not only volumetric loss or tissue descent, but also coordinated changes in dermal matrix composition, facial fat compartment behavior, muscular dynamics, and bony remodeling (19–22).

Within this broader aging framework, deterioration of dermal quality plays a particularly important role in the early stages of facial aging. Quantitative assessments based on surface analysis have shown that changes in skin texture and surface irregularity may occur independently of substantial volumetric alteration, indicating that dermal matrix degradation itself can serve as a primary driver of early aging signs (2). At the dermal level, aging is characterized by fragmentation and disorganization of collagen fibrils, loss of elastin fiber integrity, and a progressive reduction in endogenous hyaluronic acid content. These biological changes result in impaired hydration, decreased viscoelasticity, and reduced mechanical resilience of the superficial dermis (1, 3).

Fine wrinkles represent one of the earliest and most clinically relevant manifestations of these superficial dermal alterations. Unlike deep static folds, which are more closely associated with volume loss or structural descent, fine wrinkles primarily originate from cumulative microstructural failure within the papillary and upper reticular dermis. These layers depend heavily on an intact collagen–elastin network and sufficient water-binding capacity to maintain surface smoothness under dynamic conditions. Anatomical analyses indicate that this mechanism is particularly pronounced in highly mobile facial regions such as the malar and perioral areas, where repetitive motion, thin dermal architecture, and increased mechanical stress predispose the skin to early surface creasing (20, 23).

Taken together, these observations indicate that fine wrinkles are not primarily a consequence of deep volumetric deficiency, but rather reflect localized impairment of the superficial dermal matrix to withstand mechanical stress while preserving surface uniformity. Accordingly, biological rejuvenation strategies targeting fine wrinkles should prioritize restoration of dermal hydration, enhancement of viscoelastic properties, and stabilization of the superficial extracellular matrix, rather than approaches focused exclusively on volumetric augmentation or deep tissue repositioning.

### Non-crosslinked and crosslinked HA

Non-crosslinked hyaluronic acid (HA) formulations have been extensively investigated for intradermal skin quality improvement owing to their high diffusibility within the superficial dermis. In a ultrasound-based study, Bezpalko and Filipskiy evaluated two linear, non-crosslinked sodium hyaluronate gel formulations administered to the subdermal plane (6). The two formulations were both composed of non–covalently crosslinked HA, but differed in hyaluronic acid concentration and rheological properties, resulting in distinct diffusion and tissue interaction profiles.

Ultrasound measurements demonstrated that both formulations induced a measurable increase in dermal thickness following injection. However, the formulation with lower viscosity and higher diffusibility showed a more homogeneous distribution within the dermal compartment, whereas the formulation with higher viscosity exhibited relatively localized persistence at the injection plane. Despite these differences in spatial distribution, both products produced only transient improvements in skin quality parameters, with progressive attenuation during follow-up. The authors attributed this limited durability to the rapid enzymatic degradation of linear, non-crosslinked HA, regardless of concentration or viscosity.

These findings indicate that while variations in HA concentration and rheology can influence intradermal spread and short-term tissue interaction, the absence of covalent stabilization remains a fundamental limitation for sustained fine wrinkle improvement when using non-crosslinked HA formulations. Similar short-lived clinical effects have been reported in mesotherapy-based studies employing non-crosslinked HA for fine wrinkle treatment, where repeated sessions were required to maintain observable benefits (7).

To overcome the durability constraints of non-crosslinked HA, chemically crosslinked HA fillers using external crosslinking agents such as 1,4-butanediol diglycidyl ether (BDDE) or divinyl sulfone (DVS) were developed. Cui et al. demonstrated that intradermal injection of BDDE-crosslinked HA resulted in wrinkle reduction and histologic features consistent with dermal remodeling in aged human skin (8). Randomized controlled trials evaluating BDDE-crosslinked HA fillers for nasolabial folds have similarly reported superior persistence and wrinkle improvement compared with non-crosslinked formulations (9).

However, material science and biomechanical studies have shown that chemically crosslinked HA fillers exhibit increased elastic modulus, cohesivity, and structural integrity, characteristics that may restrict uniform dispersion within the superficial dermis (5, 10, 11). While advantageous for volumetric correction, these properties may limit suitability for fine wrinkles arising from the papillary and upper reticular dermis. Furthermore, *in vitro* toxicity studies have demonstrated that the type and concentration of chemical crosslinkers, including BDDE-based systems, can influence cellular responses and inflammatory potential, raising considerations for use in delicate dermal compartments (12, 13).

Collectively, prior studies delineate a clear trade-off: non-crosslinked HA formulations provide favorable superficial dermal diffusion but insufficient persistence, whereas chemically crosslinked HA fillers offer durability at the expense of optimal dispersion for fine wrinkle modulation.

Therefore, the present study employed a self-crosslinking hyaluronic acid formulation that undergoes *in vivo* crosslinking in response to local oxygen exposure and physiological pH. This approach was intended to enhance molecular persistence without reliance on external chemical crosslinkers, while maintaining adequate diffusibility for homogeneous superficial dermal distribution.

### Needle-free jet injection for skin rejuvenation

In addition to formulation characteristics, delivery method is a critical determinant of treatment outcome when targeting fine wrinkles. Conventional needle-based intradermal injections are susceptible to variability in depth, localized pooling, and inconsistent lateral dispersion, which may limit uniform treatment of superficial dermal structures. Needle-free jet injection systems have been developed to address these limitations by generating high-velocity microjets capable of reproducible intradermal penetration and lateral dispersion.

Sonographic analyses have demonstrated that jet injection can achieve consistent intradermal delivery depth, reducing operator-dependent variability (15). Comparative findings have further shown that needle-free jet delivery of polynucleotide fillers can result in comparable or improved skin rejuvenation outcomes compared with needle-based techniques, with differences in tissue distribution patterns (17). Case series and literature reviews suggest that jet-based delivery may be particularly advantageous for biostimulatory or skin-quality–focused treatments that require homogeneous superficial dermal coverage rather than focal bolus deposition (16).

These findings support the rationale for combining dermally optimized HA formulations with needle-free jet injection when the therapeutic goal is fine wrinkle modulation and skin texture improvement.

### Interpretation of study findings

In the present study, repeated intradermal delivery of a self-crosslinking hyaluronic acid formulation using a needle-free jet injection system resulted in consistent improvements in objective imaging parameters and investigator-rated clinical outcomes, with low procedural discomfort and a favorable safety profile. Taken together, these findings provide insight not only into treatment efficacy, but also into how formulation design and delivery modality jointly influence superficial dermal rejuvenation.

Objective three-dimensional imaging demonstrated reductions in wrinkle and texture scores across anatomically distinct facial regions, including the malar and pericommissural areas. These regions differ in baseline dermal thickness, mechanical stress, and dynamic movement; therefore, the parallel direction of change observed across regions suggests that the treatment effect was not region-specific or dependent on localized anatomy. Rather, the imaging results support a global modulation of superficial dermal microtopography, consistent with improved hydration, viscoelasticity, and surface uniformity, rather than focal volumetric expansion. This distinction is particularly relevant for fine wrinkle treatment, where excessive localized deposition may be undesirable.

The imaging-based findings were corroborated by blinded investigator-rated clinical assessments, which demonstrated meaningful reductions in fine wrinkle severity and improvements in skin texture. The concordance between quantitative imaging outcomes and independent clinical evaluations strengthens the interpretation that the observed changes reflect genuine dermal-level modification rather than transient surface effects. Notably, improvements in fine wrinkles were more pronounced than changes in pigmentation uniformity, indicating that the primary biological impact of the intervention was directed toward dermal structure and hydration rather than epidermal pigmentary pathways. This outcome pattern aligns with the intended mechanism of action of hyaluronic acid–based dermal modulation and further differentiates this approach from treatments targeting melanogenesis or epidermal turnover.

When compared with prior hyaluronic acid–based rejuvenation studies, the present findings highlight several points of distinction. Non-crosslinked HA formulations have previously been shown to improve skin hydration and texture, but their effects are often short-lived due to rapid degradation (6, 7). Conversely, conventional crosslinked HA fillers provide greater persistence but may exhibit limited suitability for uniform superficial dermal dispersion because of their rheological properties and cohesivity (5, 10, 11). In contrast, the self-crosslinking HA formulation used in this study appears to occupy an intermediate functional space, offering enhanced tissue residence without the stiffness or bolus-forming tendency associated with heavily crosslinked fillers. The observed improvements in wrinkle and texture scores, achieved without evidence of localized volumization or irregular surface contour, support this conceptual positioning.

The delivery modality further differentiates the present approach from existing rejuvenation strategies. Previous studies of needle-free jet injection systems have demonstrated improved depth consistency and lateral dispersion compared with manual needle-based intradermal injections (15–17). In the context of fine wrinkle treatment, where therapeutic targets are confined to the superficial dermis, uniform distribution may be as critical as formulation composition. The consistent imaging and clinical outcomes observed across multiple facial regions in this study suggest that jet-based delivery contributed to reproducible intradermal dispersion, minimizing focal pooling and depth variability that can occur with conventional techniques.

Procedural pain scores remained low to moderate across repeated treatment sessions, without evidence of cumulative discomfort. This finding is clinically relevant because superficial dermal rejuvenation protocols typically require multiple treatment sessions to achieve and maintain optimal outcomes. The absence of escalating pain suggests that the needle-free jet system may facilitate repeated intradermal delivery with acceptable tolerability, supporting its feasibility for multi-session treatment regimens.

Safety outcomes further contextualize the clinical applicability of this approach. Adverse events were limited to transient, localized reactions commonly associated with intradermal procedures, and no delayed inflammatory responses or serious complications were observed. This favorable safety profile may reflect both the absence of conventional chemical crosslinkers in the HA formulation and the avoidance of tissue cutting associated with solid needles. Compared with reports of inflammatory nodules or delayed reactions associated with certain crosslinked filler formulations, the present findings suggest that this combination of formulation and delivery may be particularly suited for superficial dermal applications.

Overall, the results of this study suggest that fine wrinkle improvement can be achieved through a strategy that prioritizes superficial dermal modulation rather than volumetric correction. By integrating a self-crosslinking hyaluronic acid formulation with needle-free jet injection, the present approach differs from conventional filler-based rejuvenation paradigms and offers a targeted method for improving fine wrinkles and skin texture with reproducible outcomes, acceptable tolerability, and a favorable safety profile.

### Limitations and future research

This study has limitations, including its single-arm, non-randomized design and lack of a comparator group, which precludes direct comparison with needle-based injection or alternative HA formulations. In particular, because no control group received saline or non-crosslinked HA using the same needle-free jet injection device, the specific contribution of the self-crosslinking HA formulation cannot be fully separated from the mechanical effects of jet delivery itself. The follow-up period was limited to 12 weeks, and the long-term durability of observed improvements remains to be determined. In addition, while objective three-dimensional imaging provided quantitative assessment of wrinkle and texture changes, histologic or biomechanical validation of dermal remodeling was not performed. Furthermore, the generalizability of the study findings is limited by the cohort’s specific demographic characteristics. As the study exclusively enrolled Asian women aged 40–55 years with Fitzpatrick skin types III and IV, the results may not be directly generalizable to male patients, other age groups, or individuals with different ethnic backgrounds or skin phototypes.

Additionally, there are inherent limitations to the needle-free jet delivery method itself. Although this technique is intended for superficial dermal delivery, it may not be appropriate for all types of wrinkles in all patients. Deeper static rhytides, wrinkles associated with marked volume loss, severe skin laxity, or heavily photodamaged and fibrotic skin may respond less adequately to superficial jet-based delivery alone and may require alternative or multimodal treatment approaches.

Future studies incorporating randomized controlled designs, extended follow-up periods, and direct comparisons between delivery techniques and formulation types will be necessary to further elucidate the relative contributions of material properties and injection modality. Integration of ultrasound or biomechanical assessment may also provide additional insight into dermal changes underlying fine wrinkle improvement. Future research should also aim to validate these findings across more diverse patient populations and evaluate treatment outcomes according to wrinkle severity, facial region, baseline skin thickness, and skin phototype to better define optimal patient selection criteria.

## Conclusion

This study suggests that needle-free jet injection of a self-crosslinking hyaluronic acid formulation may induce measurable improvements in fine wrinkles and skin texture, consistent with modulation of superficial dermal properties. By addressing both formulation stability and intradermal distribution uniformity, this approach appears to represent a targeted rejuvenation strategy for fine wrinkle improvement that is distinct from traditional volumetric filler paradigms.

## Data Availability

The raw data supporting the conclusions of this article will be made available by the authors, without undue reservation.
